# Presumed sclerotomy site bleeding inflowing into the anterior chamber after the removal of a 23-gauge microcannula in 23-gauge sutureless vitrectomy

**DOI:** 10.4103/0301-4738.71709

**Published:** 2010

**Authors:** Dong Heun Nam, Sang Chul Yoon, Dae Yeong Lee, Hee Jin Sohn

**Affiliations:** Department of Ophthalmology, Gachon University Gil Hospital, Incheon, South Korea

**Keywords:** Bleeding, sclerotomy site, sutureless vitrectomy

## Abstract

We experienced two cases of the influx of the sclerotomy site bleeding into the anterior chamber during 23-gauge sutureless vitrectomy for pseudophakic rhegmatogenous retinal detachment. Soon after the removal of a 23-gauge microcannula at the end of the surgery, presumed sclerotomy site hemorrhage was rapidly fluxed into the anterior chamber. The anterior chamber bleeding might come from the sclerotomies rather than from episcleral vessels. The posterior pressure in the gas-filled pseudophakic eye might have pushed the sclerotomy site bleeding into the anterior chamber. We could not find any vitreous hemorrhages. The hemorrhage within the anterior chamber spontaneously absorbed within 14 days.

Transconjunctival sutureless vitrectomy has gained popularity in recent years. The use of 25-gauge or 23-gauge instruments showed many advantages over the conventional 20-gauge vitrectomy system, and there were many reports on the safety and efficacy of 25-gauge or 23-gauge vitrectomy for a variety of vitreoretinal diseases.[1,2] However, the complications involving those small-gauge instruments remain problematic.[3–5] We experienced presumed sclerotomy site hemorrhage inflowing into the anterior chamber following the removal of the 23-gauge microcannula in 23-gauge sutureless vitrectomy.

## Case Reports

### Case 1

A 57-year-old man with diabetes was diagnosed with inferotemporal pseudophakic rhegmatogenous retinal detachment with a retinal tear at the 11 o’clock position in the right eye. Best-corrected visual acuity (BCVA) was 20/200 and the axial length was 22.86 mm.

**Figure 1:**
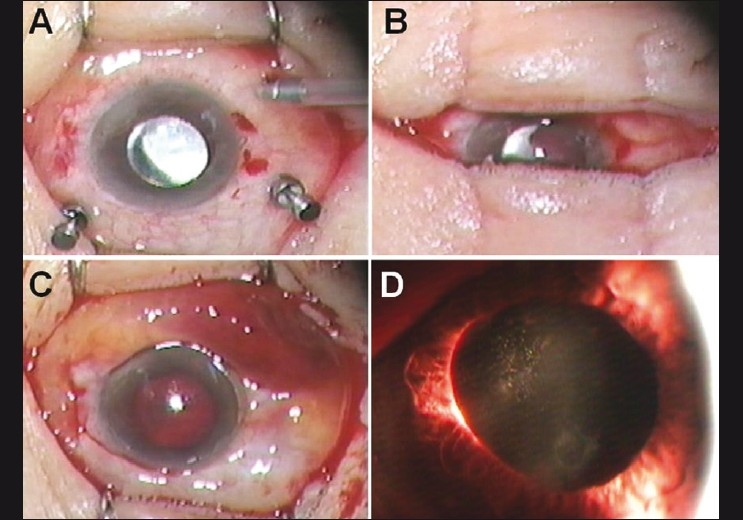
Sequential intraoperative photographs (A–C). (A) Microscopic overview showing no hemorrhagic sign at the end of vitrectomy. (B) About 15 s after the removal of the cannulae, hemorrhage was found inflowing into the anterior chamber from the inferotemporal area of the limbus. (C) Anterior chamber hemorrhage, severe subconjunctival hemorrhages and chemosis in the inferotemporal sclerotomy site. (D) Slit-lamp photograph at postoperative 14 days shows that the hemorrhage was completely absorbed

Vitreous surgery was carried out using a 23-gauge vitreous cutter driven by a vitrectomy unit (Associate 2500, DORC International, The Netherlands) and the DORC two-step system (DORC International). The procedure was started with a scleral tunnel incision radial to the limbus, as Eckardt described.[2] During the surgery, there were no specific events such as hemorrhages. Endolaser photocoagulation and air-gas (12% C_3_F_8_) exchange were performed. At the end of the surgery, the three microcannulae were withdrawn from the scleral tunnel and the immediate postoperative tactile intraocular pressure (IOP) felt normal [Fig. 1A]. About 15 s after removal of the cannulae, the anterior chamber hemorrhage was noticed [Fig. 1B]. It was rapidly inflowing from inferotemporal area of the chamber into the whole space. In addition, there were severe subconjunctival hemorrhages and chemosis in the inferotemporal sclerotomy site [Fig. 1C]. Surgery was completed without any additional procedure. Postoperatively, we could not find any vitreous hemorrhages and the sclerotomy was well closed. The anterior chamber hemorrhage and subconjunctival hemorrhage were completely absorbed within 14 days [Fig. 1D] and the IOP maintained was 14 mmHg. Six months after the surgery, BCVA was 20/40 and fundus examination revealed a flat retina.

### Case 2

A 46-year-old man without diabetes was diagnosed with superior pseudophakic rhegmatogenous retinal detachment with a retinal tear at the 12 o’clock position in the right eye. BCVA was 20/100 and the axial length was 24.05 mm.

**Figure 2:**
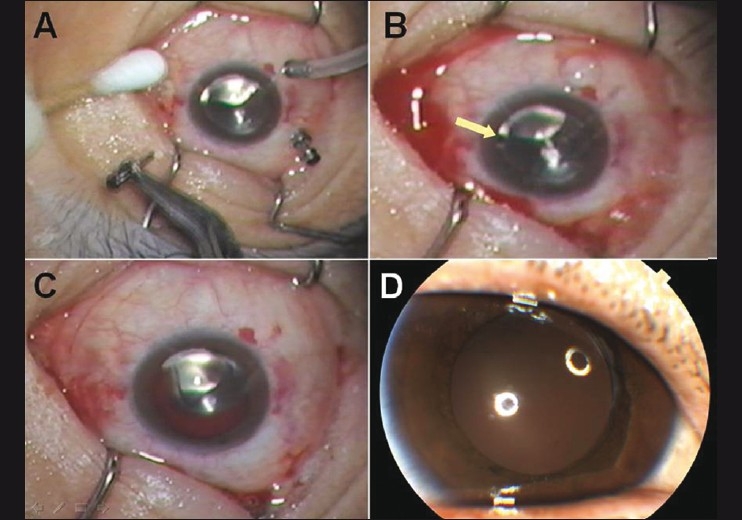
Sequential intraoperative photographs (A–C). (A) No hemorrhagic sign at the end of vitrectomy. (B) About 20 s after the removal of the cannulae, hemorrhage starts inflowing into the anterior chamber from the superonasal area of the limbus (yellow arrow). (C) Anterior chamber hemorrhage. (D) Slit-lamp photograph at the postoperative 7 days showing a clear anterior chamber

Vitreous surgery was carried out using a 23-gauge vitreous cutter and the DORC two-step system. During the surgery, there were no specific events. Endolaser photocoagulation and air-gas (12% C_3_F_8_) exchange were performed. At the end of the surgery, the three microcannulae were withdrawn from the scleral tunnel and the immediate postoperative tactile IOP felt normal [Fig. 2A]. About 20 s after the removal of the cannulae, the anterior chamber hemorrhage was noticed [Fig. 2B]. It was slowly inflowing from the superonasal area of the chamber [Fig. 2C]. Meanwhile, there was neither subconjunctival hemorrhage nor chemosis in the superonasal sclerotomy site. Surgery was completed without any additional procedure. Postoperatively, we could not find any vitreous hemorrhages and the sclerotomy was well closed. The anterior chamber hemorrhage was completely absorbed within 7 days [Fig. 2D] and the IOP maintained was 17 mmHg. Six months after the surgery, BCVA was 20/40 and fundus examination revealed a flat retina.

## Discussion

The scleral incision with the 23-gauge or 20-gauge stiletto blade could frequently lead to bleeding from episcleral vessels or sclerotomies. Even though it is only mild bleeding, diathermy has usually been used in conventional 20-gauge vitrectomy. However, it is unavailable in transconjunctival sutureless vitrectomy. Experienced surgeons have noticed that the placement of the microcannula within the tunnel always resulted in the stoppage of the bleeding and the withdrawal of the cannula sometimes resulted in minor subconjunctival bleeding in the area of sclerotomy.[2] In a study of endoscopic observation, Koch *et al*.[6] reported sclerotomy site microbleeding, which stopped spontaneously.

In this study, both patients had two-step 23-gauge sutureless vitrectomy and gas tamponade for pseudophakic rhegmatogenous retinal detachment. Intraoperatively, we could not find any intraocular or periocular hemorrhage except for minor subconjunctival bleeding in the area of sclerotomy. Soon after the removal of the microcannulae at the end of the surgery, we noticed significant subconjunctival bleeding just in one eye. Furthermore, the hemorrhage inflowing into the anterior chamber is considered as intraocular hemorrhage. Therefore, we suppose that the anterior chamber bleeding might come from the sclerotomies rather than from episcleral vessels. The posterior pressure in the gas-filled pseudophakic eye might have pushed the sclerotomy site bleeding into the anterior chamber.

Although anterior chamber bleeding after sutureless vitrectomy is a rare complication, clinicians should be aware of this inevitable adverse event when preparing patients for pseudophakic rhegmatogenous retinal detachment surgery.
